# The Comet Assay for the Evaluation of Genotoxic Potential of Landfill Leachate

**DOI:** 10.1100/2012/435239

**Published:** 2012-04-30

**Authors:** Kamila Widziewicz, Joanna Kalka, Magdalena Skonieczna, Paweł Madej

**Affiliations:** ^1^Institute of Water and Wastewater Engineering, Silesian University of Technology, Akademicka Street 2A, 44-100 Gliwice, Poland; ^2^Environmental Biotechnology Department, Silesian University of Technology, Akademicka Street 2A, 44-100 Gliwice, Poland; ^3^Institute of Automatic Control, Silesian University of Technology, Akademicka Street 2A, 44-100 Gliwice, Poland

## Abstract

Genotoxic assessment of landfill leachate before and after biological treatment was conducted with two human cell lines (Me45 and NHDF) and *Daphnia magna* somatic cells. The alkali version of comet assay was used to examine genotoxicity of leachate by DNA strand breaks analysis and its repair dynamics. The leachate samples were collected from Zabrze landfill, situated in the Upper Silesian Industrial District, Poland. Statistically significant differences (Kruskal-Wallice ANOVA rank model) were observed between DNA strand breaks in cells incubated with leachate before and after treatment (*P* < 0.001). Nonparametric Friedman ANOVA confirmed time-reliable and concentration-reliable cells response to leachate concentration. Examinations of chemical properties showed a marked decrease in leachate parameters after treatment which correlate to reduced genotoxicity towards tested cells. Obtained results demonstrate that biological cotreatment of leachate together with municipal wastewater is an efficient method for its genotoxic potential reduction; however, treated leachate still possessed genotoxic character.

## 1. Introduction

During the independent laboratory studies genotoxicity of landfill leachate has been proved [1–4]. Landfill leachate is generated due to the infiltration of rainwater through the waste mass and due to the wastes biodegradation. Leachate can penetrate into groundwater and migrate for considerable distances causing environmental contamination. As a result leachate compounds can be accumulated in the successive links of the food chain or in long-term exposure by human beings [4]. Evaluation of the toxic and genotoxic potential of landfill leachate towards organisms is acquiring a particular significance especially in the case of a constant exposure. The genotoxic influence can lead to the changes including one generation (genome damages in somatic cells) or to the long-term effects (genome damages in germ cells). That can result in reduced fertility of the populations inhabiting aquatic environment, biodiversity depletion, or, in extreme cases, in total extinction. Assessing the ecosystem response towards genotoxic factors is difficult, therefore more often in the water monitoring, apart from physicochemical analyses and acute toxicity tests, an individual species reaction on molecular level is being examined. *In vitro* genotoxicity tests have especially gained increasing popularity as a tool supporting environmental risk assessment. DNA damage levels relatively early provide information about the genotoxic potential of the environmental compartment, enabling taking preventive strategies. A popular method for measuring DNA damage is single cell gel electrophoresis (SCGE) known as comet assay. This technique is widely spread as a simple, sensitive, and fast tool for the evaluation of DNA damages and its repair in single cells [5]. SCGE can be a good measure of genotoxic potential even in early stages of exposure. A frequently used approach is carrying SCGE on the same population after removing genotoxic intervention with an aim of examining DNA repair mechanisms, which allows to estimate a possible adaptation to low concentrations of the genotoxic factor. Comet assay combines the simplicity of biochemical detection techniques of DNA double-strand breaks (DSB), DNA single-strand breaks (SSB), alkali labile sites (ALS) such as apurinic/apyrymidinic (AP) sites, and DNA-DNA and DNA-protein cross-links with typical cytotoxicity tests.

In reference to other genotoxicity tests, for example, chromosome aberration, sister-chromatid exchange, alkaline elution or micronuclear test, the advantages of the comet assay are:

- evaluation of damages in all types of cells,
- high sensitivity (one DNA break per 1010 Da of DNA can be detected),
- small number of cells necessary for analysis (10 000 cells per sample),
- flexibility in choosing cells for the examinations (both proliferating and nonproliferating),
- low costs,
- simplicity and relatively short time of the analysis,
- small amount of substances being tested,
- reliable statistical analysis,
- possibility of the evaluation of different DNA damages [6, 7].

The comet assay is based on the principle of quantifying the amount of denatured DNA fragments migrating out of the nuclei during electrophoresis. During separation across an applied electric field, DNA remaining in the place of the flooded cell, partly anchored to residual nucleus structures, is migrating towards the anode with the speed adequate to its fragmentation. The DNA migration speed in agarose is directly proportional to its damage degree. The image obtained by this procedure look like a “comet” with a distinct head and tail constitute of relaxed loops and damaged DNA fragments.

The aim of the study was determination of genotoxic potential of landfill leachate sampled from “old,” reclaimed, municipal landfill. Leachate samples were the subject of treatment in laboratory model of activated sludge. The efficiency of treatment was evaluated at the base of chemical parameters and genotoxic potential reduction.

## 2. Materials and Methods

### 2.1. Leachate Origin

The examinations were performed with leachate collected from the old quarter of solid waste landfill in Zabrze (Poland), which was already closed and subjected to the recultivation. Leachate from quarter was collected by sewage collection system in the equalization basin (internal volume 160 m^3^), from where it was taken for further examinations in two-week intervals (IX 2009-V 2010 period). Leachate characteristic is presented in Table 1.

### 2.2. Laboratory Unit

The reactor for leachate treatment was working as the classical biological A2/O system. The system was consisted of three serially connected chambers with different oxygen conditions: anaerobic (vol. 2 dm^3^), anoxic (vol. 5 dm^3^), and aerobic (vol. 7 dm^3^) and the secondary settling tank. Moreover, the reactor was equipped with external recirculation system (approaching 100% of influent) and internal recirculation of wastewater into the anoxic chamber (250% of influent). The sludge concentration in the reactor was kept on the level of 2.5 g SS/dm^3^ and the influent flow rate was 1.7–2.5 dm^3^/d. At the beginning of the experiment reactor was fed with a constant flow of raw municipal wastewater. Biomass was gradually acclimated to increasing landfill leachate concentration (0–100% v/v). Genotoxicity examinations were conducted in the last phase of reactor operation, when undiluted leachate were treated. Influent and effluent from the laboratory system were kept refrigerated until use (−40°C). Before analysis, wastewater samples were defrosted and filtered through membrane filters (pore size 0.2 *μ*m). Influent and effluent samples were also examined for basic physicochemical parameters (pH, conductivity, COD, BOD_5_, N_tot_, and N-NH_4_).

### 2.3. Cell Cultures and Damages Induction

Tests were conducted with two human cell lines (Me45—Human melanoma; NHDF—normal human dermal fibroblasts) cultured in DMEM medium (Sigma), supplemented with 10% fetal calf serum (FCS) and 1% gentamicin. Cells were obtained from the Institute of Oncology in Gliwice, Poland. Tests were also conducted on *Daphnia magna* somatic cells from the Department of the Environmental Biotechnology, Silesian University of Technology. Crustaceans came from healthy culture (i.e., did not demonstrate signs of stress like high mortality, delay in the production of the first brood, discoloured animals, etc.). The comet assay allowed for quantitative determining number of the DNA strand breaks after exposition to leachate concentrations 0.1%, 1%, and 10% (v/v) before and after biological treatment. Non exposed cells constituted the control group.

### 2.4. Comet Assay

#### 2.4.1. Cell Lines Me45 and NHDF

Me45 and NHDF cells were trypsinized (10x diluted) and washed once with DMEM (Dulbecco modified eagle's medium), harvested by centrifugation (900 g, 2 minutes) and suspended at a density ~8 × 10^5^ cells/mL. In the next step Me45 and NHDF cells were divided to 4 plates described as follows: control group: 0.1%, 1%, and 10 % (v/v) concentrations of wastewater. Cells were exposed for 15 minutes for the indicated wastewater concentrations in 3% CO_2_ atmosphere. Leachate concentrations were selected at the base on earlier acute toxicity tests (unpublished data). The induction was stopped by suspending cells in the fresh medium. 50 *μ*L of suspension was taken for comet assay immediately after 0, 15, 30, 60, 120, and 180 min repair time and transferred to Eppendorf tubes. Cells were mixed with 100 *μ*L of 1 % LMP agarose (Sigma) and placed on microscope slides covered with 0.5% NMP agarose (Sigma). The gel was solidified for 10 min. on ice. Cells were then lysed for 60 min in 2.5 M NaCl, 100 mM EDTA, 10 mM Tris/HCl, pH 7.5, 1% Triton X-100, denaturation was for 20 min in 300 mM NaOH, 1 mM EDTA, pH 13. Before electrophoresis, slides were incubated in a jar containing electrophoresis buffer (300 mM NaOH, 1 mM EDTA, pH > 13) for 15 minutes for DNA unwinding and the expression of alkali-labile damage. Electrophoresis was in the same buffer for 20 min at 1 V/cm [8]. After electrophoresis the slides were neutralized for 5 min in 0.4 M Tris/HCl buffer, pH 7.5, and stained with 20 *μ*L (2 *μ*g/mL) ethidium bromide.

#### 2.4.2. *Daphnia magna*

*Daphnia magna* organisms were exposed to sublethal leachate concentrations (0.1%, 1.0%, 10% v/v) before and after biological treatment. For leachate dilution aerated tap water was used. Non exposed organisms constituted the control group. After 48 hours, 10 organisms per sample were taken for further analysis. Treated organisms were suspended in 1 mL of PBS solution containing 20 mM (EDTA) and 10% (DMSO), and in second step subjected to homogenization. Further procedure was similar like in case of human cellular lines; however, time of lysis was shortened to 20 minutes.

### 2.5. Data Analysis

The DNA migration was measured using Comet Score Freeware v1.5 (TriTek Corporation). The images were taken with the camera connected to fluorescence microscope Axio Imager 2, Carl Zeiss Company (400x, 590 nm filter). Fifty cells were measured for DNA migration on each slide. For genotoxicity assessment Olive tail moment defined as the product of DNA in the tail and the mean distance of migration in the tail was used, according to Olive et al. [5]. Testing normality of distributions was based on Shapiro-Wilk statistics. Comparison between leachate genotoxicity before and after treatment was done with the use of Kruskal-Wallis ANOVA rank model. A trend analysis together with nonparametric Friedman ANOVA allowed dose-response relationships (OTM versus dose of leachate, OTM versus repair time) to be investigated.

## 3. Results and Discussion

Leachate is characterized by high concentrations of COD, BOD_5_, and organic matter. Because of refractory organics content, high N-NH_4_ load, leachate is typically resistant to biological treatment processes. Discharging leachate into surface water without any treatment effect in high pollution of the receiver. The present work examined possibility of leachate genotoxicity reduction by its co-treatment with municipal wastewaters. 

The physicochemical characteristics of the raw and co-treated leachate are shown in Table 1. COD and BOD_5_ values demonstrate that after treatment they were reduced by 53% and 97%, respectively. The BOD_5_/COD ratio for leachate before and after treatment was, respectively, 0.33 and 0.02 which reveal the presence of nonbiodegradable fraction of organic matter. This proportion is smaller than typical ratio recommended for biologically treated wastewater (0.4 ÷ 0.6) [9, 10]. Efficiency removal for N_total_ and N-NH_4_ was 83.7% and 97%, respectively. Conductivity and pH were not significantly changed after treatment. 

In the present study we proposed an integrated strategy to evaluate the genotoxicity of the leachate by connecting chemical analyses together with *in vitro *and* in vivo* bioassays. Results obtained by Shapiro-Wilk *W*-test (Tables 2 and 3), show that these data distributions are generally non-Gaussian, even after logarithmic transformation as recommended in the literature, which precludes the use of parametric tests for further statistical analysis [11].

The data demonstrated that untreated leachate samples were characterized by higher genotoxicity in comparison to leachate after treatment. In all untreated samples a significant increase of Olive tail moment (OTM) with the increase of leachate concentration was observed (Table 3). The differences in OTM for control samples could also be noticed. We assumed that differences in cell preparations and/or factors like, UV, incubation temperature, and so forth, can be source of variation in background DNA damages. The DNA breaks can vary as a function of procedure conditions, genetic background of the cell line, number of passages, and expression levels of the DNA repair enzymes. Therefore it is essential to perform always an assay with negative control and all obtained results compare to the control value.

**Figure 1:**
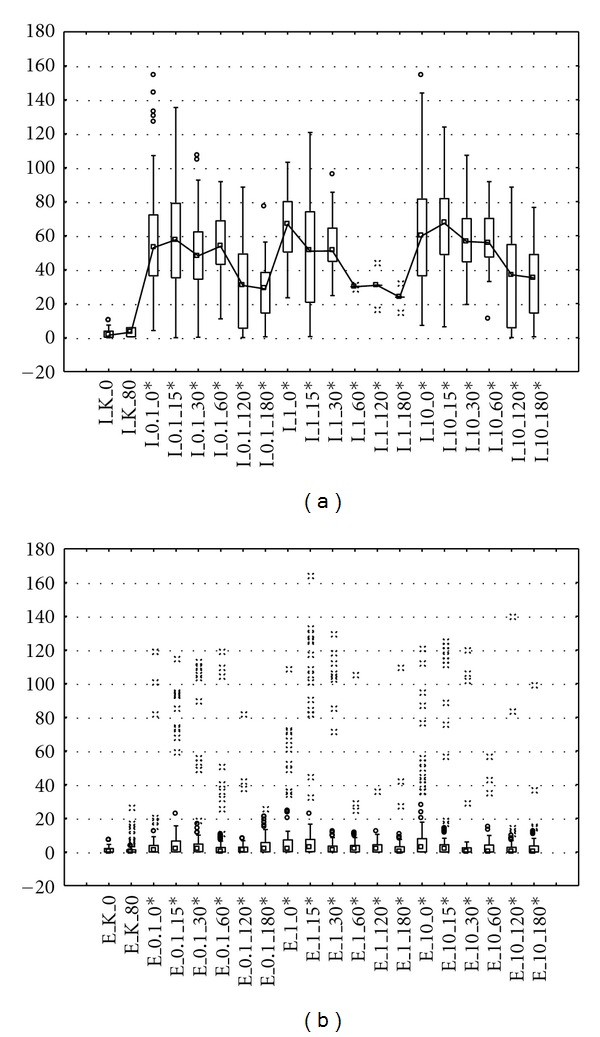
Comets observed for Me45 cells treated by 0.1%, 1%, and 10% of the leachate ((a) influent, (b) effluent). Each line corresponds to 50 comets measured on one slide; the probability in the Kruskal-Wallis test for the difference between influent and effluent in parentheses: *P* < 0.001 (∗).

**Figure 2:**
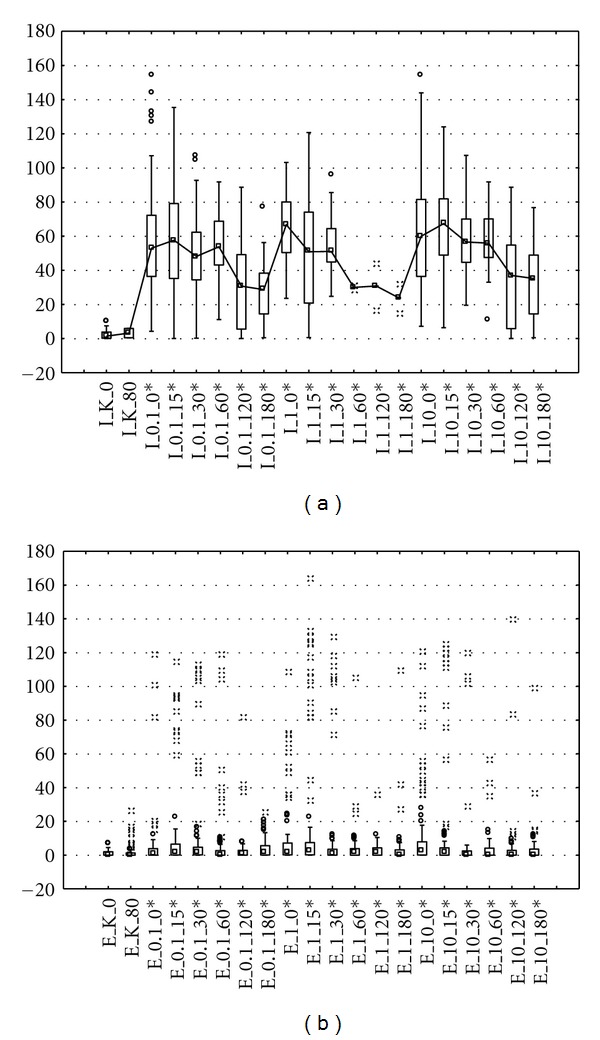
Comets observed for NHDF cells treated by 0.1%,1%, and 10% of the leachate ((a) influent, (b) effluent). Each line corresponds to 50 comets measured on one slide; the probability in the Kruskal-Wallis test for the difference between influent and effluent in parentheses: *P* < 0.001 (*).

Median and 75th percentile were applied as suitable measures for highly skewed Olive Tail Moment distributions. Median values for leachate before treatment were significantly higher than after treatment (*P* < 0.001, Kruskal-Wallis ANOVA rank model) (Figures 1 and 2). We conclude that analysis of median comet metrics from experiments at different exposure rate levels is certainly an efficient way to statistically demonstrate a leachate genotoxic effect. This approach is similar to that suggested by Duez [12]. Comparison of Figure 1(a) with Figure 1(b) as well as Figure 2(a) with Figure 2(b) showed that there was difference in baseline median OTM values between the leachate before and after treatment, which in most cases can be confirmed through visual evaluation of boxplot graphs. In the notched boxplot, if two boxes notches do not overlap, this is strong evidence, their medians differs. Leachate in 0%, 1%, 1%, and 10% concentrations produced significant amounts of damage when compared with the control. There was a clear dose-dependent response following either treatment for both Me45 and NHDF cell lines with increasing values of OTM accompanying increasing leachate concentrations. 

The treatment results expressed by OTM values for NHDF line ranged from 0.2 for lowest exposure dose, 0.1% leachate of effluent after 180 min time, to 67.8 in highest 10% influent concentration after 0 min (Figures 2(a) and 2(b)). The same situation relates to significant heterogeneity of DNA damage for Me45 line where OTM ranged from 3.06 for cells exposed to 10% effluent concentration after 120 min to 65.35 for 1% influent concentration after 0 min (Figures 1(a) and 1(b)). In most cases number of damages was proportional to a growing concentration of leachate (Tables 1 and 2). Genotoxic potential of leachate, especially for leachate before treatment in highest concentration, may be lowered because of the marked presence of big hedgehog comets that did not allow properly damages classification and consequently the genotoxicity determination. The limitations in apoptotic cells classification were also noted by other authors [12].

Nonparametric Friedman ANOVA was used to evaluate the differences in time-reliable cells responses for different leachate concentrations. Friedman statistics is an alternative for repeated-measures regression analysis. In this case it allows simultaneous testing and modeling two variables (independent variable-concentration and variable time). For both cell lines variables concentration and time, were significant predictors of OTM. Kendall Tau correlation coefficient was used to find the association between these two measured quantities. We observed strong statistical dependence (*P* < 0.05. Friedman test) between time and concentration in all samples, excluding Me45 cells response to leachate after treatment (Table 4). 

**Figure 3:**
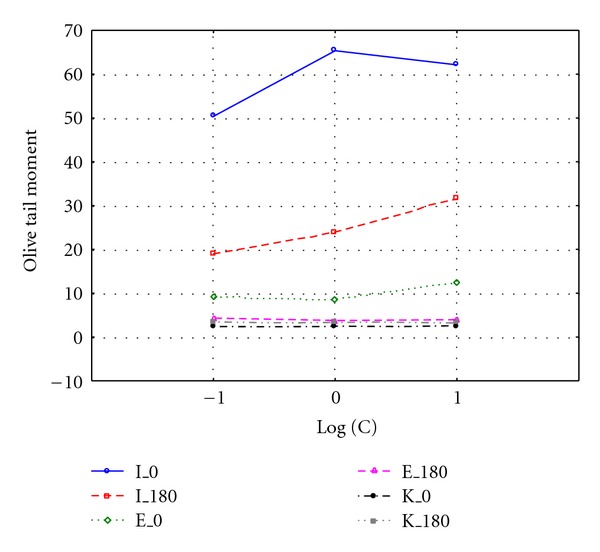
Olive tail moment in Me45 cell line exposed to 0.1%, 1%, and 10% leachate concentration before (I) and after (E) treatment; K: control samples.

**Figure 4:**
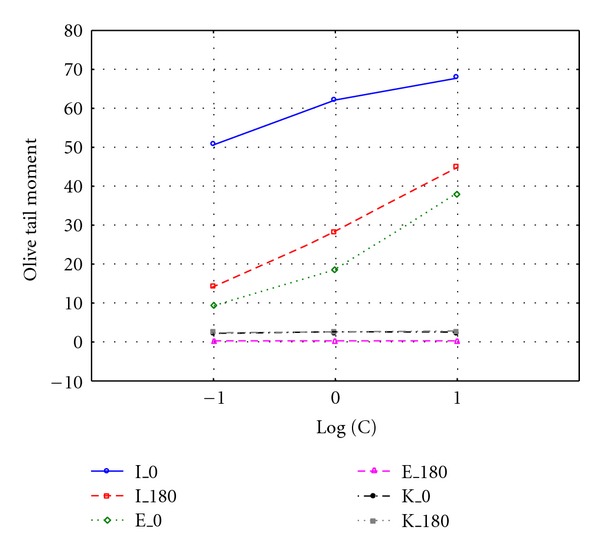
Olive tail moment in NHDF cell line exposed to 0.1%,1%, and 10% leachate concentration before (I) and after (E) treatment; K: control samples.

Significant difference was observed when comparing OTM values after 0 and 180 minutes with logarithm of exposure dose (Figures 3 and 4). Cells exposed to untreated leachate showed higher DNA damage, slower repair, and higher residual unrepaired damage than those after treatment. Median values of OTM were much smaller for the undamaged control cells than for the damaged cells, and were decreasing with increasing repair time. Cultured cells exposed to leachate respond with an immediate increase in DNA strand breaks (time 0 minutes), with gradually disappear during repair time, which also can be visualized by the line connecting box plots on Figures 1(a) and 2(a). The experimental data of DNA repair dynamic in exposed cells can be fitted to exponential curves, which means that repair enzymes are more efficient just after exposure [13]. OTM for cells exposed to surrogate leachate, even after 180 min repair time were significantly higher (Kruskal-Wallis test, *P* < 0.05) than OTM values for control group, which may suggest that non-treated leachate caused irreversible changes on molecular level. Already after 60 minutes we observed complete DNA repair in cells exposed to treated leachate. It means that although leachate co-treatment in biological system did not remove genotoxicity of treated leachate, it was reduced significantly and DNA damage repair mechanisms were accelerated.

The dose-response effect was also proven by *in vivo* studies using an aqueous crustacean *Daphnia magna*. In this study *Daphnia magna* was exposed to leachate and DNA damage was assessed in cells isolated from it.

**Figure 5:**
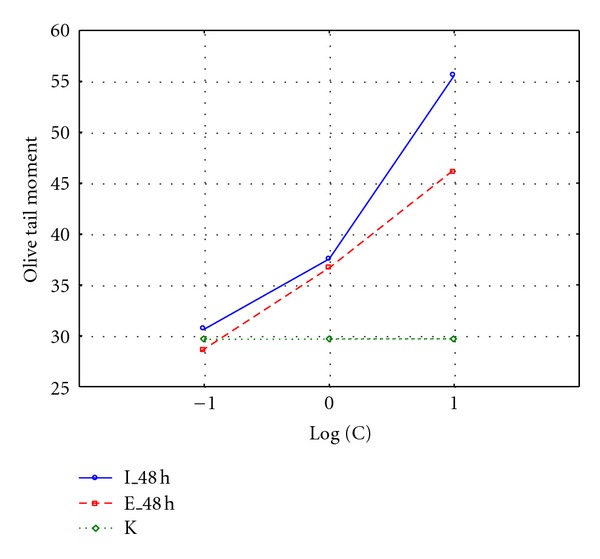
Olive tail moment in *Daphnia magna* cells exposed to 0.1%, 1%, and 10% leachate concentration before (I) and after (E) treatment.

 As shown on Figure 5, all of the concentrations of tested leachate caused an increase in the OTM values in relation to the control group. The DNA damages showed increase in relation to rising concentration of the leachate, for example, OTM in highest concentration 10% of leachate before treatment was 56 and for leachate after treatment was 46. The highest OTM level was noted for 10% of leachate concentration in relation to control group. In the analysis of genotoxic effects in *Daphnia magna* cells leachates presented similar results, suggesting that even after treatment, the leachate exhibit genotoxic potential. Taking whole organisms for experiments did not allow for the establishment of clones characterized by little genetic variability like in human cell lines case. Probably it was the main cause of the differences between OTM in control group between human and *Daphnia magna* cell lines. The data obtained from *in vivo* studies did not allow for a full statistical analysis.

## 4. Conclusions

Obtained results demonstrate that biological co-treatment of leachate together with municipal wastewater is an efficient method for its genotoxic potential reduction. The alkaline comet assay results obtained by *in vitro* as well as *in vivo* studies suggests that leachates before biological treatment provoke higher-level consequences then after treatment. The comet assay parameters significantly increased already after 15 min of exposure time in human cell lines as well as after 48 h in case of *Daphnia magna* cells in relation to unexposed control samples. Genotoxicity of leachate reflected by OTM measurement in human and *Daphnia magna* cells after treatment was significantly lower (*P* < 0.001) than before treatment. Similar pattern was observed with other biotests; authors observed significant decline of acute toxicity with *Daphnia magna* after leachate biological treatment [14].

The comet assay results revealed higher repair capacity after leachate treatment. The untreated leachate showed genotoxicity in tested human cells even after 180 minutes of the repair time, indicating the persistence of genotoxic substance, while biological treatment allowed for reduction of the genotoxic factors present in the effluent. It should be; however, noted that biological treatment of landfill leachate from “old” waste landfill did not eliminate genotoxic potential of treated medium. It is therefore concluded that advanced processes should be implemented in order to prevent natural environment against genotoxic factors. Repair analysis indicated also that background level of DNA damages in human and *Daphnia magna* control cells differs, in relation to exposure time and cells origin. 

The A2O system was shown to be efficacious in decreasing the levels of COD, BOD_5_, N_og_, and N-NH_4_. This reduction was correlated with genotoxicity decrease. The possible explanation for genotoxicity reduction after treatment can be biochemical reactions occurring in the biological process like xenobiotics sorption on sewage sludge (some xenobiotics having strongly hydrophobic properties like PCB, PAH, or heavy metals) [15]. Presented data are in agreement with the results obtained by other researchers [3, 4, 16] although the compatibility between physicochemical parameters reduction and their eco- and genotoxicity cannot be combined only with leachate chemical properties. It has been noted that other factors like age of the landfill, seasonal variations [4], solid waste stabilization [17], and others have a significant effect on genotoxic potential of leachate [9]. In our study the intercellular variability in DNA damage differs between *in vitro* and *in vivo* studies as well as between human cell lines showing the need for multifactorial environmental monitoring on different levels of molecular complexity. Many research demonstrated, that landfill leachate can influence the genetic stability of single cells; however, these assumptions are not sufficient for concluding about entire organisms response. It is commonly known that toxicity of leachate is a sum of possible antagonistic, synergistic effects of its numerous contaminants. *In vitro* models reflected the genotoxicity of leachate integrating the biological effects of all its compounds [18–20]. This work once again indicated the importance of implementing into environmental monitoring different short-term *in vitro* and *in vivo* bioassays, which together with classical physicochemical analysis can regulate wastewater and landfill leachate risk assessment. 

## Figures and Tables

**Table 1:** Physicochemical parameters of leachate.

Parameter	Unit	Range	Mean ± SD	Percentile 80/100	Range	Mean ± SD	Percentile 80/100	Reduction (%)
			Influent (before treatment)			Effluent (after treatment)		
pH	—		7.5–8.2			7.0–7.7		

*κ*	mS/cm	3.3–4.1	3.5 ± 0.3	3.6	3.8–3.9	3.9 ± 0.2	4	—
COD	mg/dm^3^	381–435	403 ± 19	418	160–233	190 ± 23	198.9	53
BOD_5_	mg/dm^3^	120–150	134 ± 11	142.1	0–7	4 ± 2.6	5	97
N_total_	mg/dm^3^	37.7–74.8	66.0 ± 16.4	68.6	6.8–18.2	10.7 ± 5.5	18.2	84
N-NH4	mg/dm^3^	26.1–51.9	43.3 ± 10.5	51.7	0.5–2.6	1.4 ± 1.0	2.3	97

**Table 2:** Average Olive moment mean values ± (SD) of the amount of DNA damage of Me45 line before and after leachate treatment. Shapiro-Wilk statistic and its probability for testing distributions normality. Normal distribution before transformation (∗). Normal distribution after log_10_ transformation (∗∗).

Time (min)	Before treatment			After treatment		
	Olive moment			Olive moment		
	Me45_0.1%	Me45_1%	Me45_0.1%	Me45_0.1%	Me45_1%	Me45_10%
control_0		2.50 ± 2.7			1.3 ± 2.15	
		*P* < 0.0001			*P* < 0.0001	

control_180		3.19 ± 3.12			2.18 ± 4.9	
		*P* = 0.0239			*P* < 0.0001	

0	55.91 ± 28.07	65.35 ± 19.79	62.11 ± 33.82	9.17 ± 25.35	8.71 ± 17.95	12.31 ± 24.4
		(∗)				
	*P* = 0.0004	*P* = 0.2683	*P* = 0.0463	*P* < 0.0001	*P* < 0.0001	*P* < 0.0001

15	54.59 ± 33.05	51.24 ± 34.11	65.57 ± 25.11	9.91 ± 23.61	14.56 ± 33.70	10.03 ± 27.52
			(∗)			
	*P* = 0.0016	*P* = 0.0075	*P* = 0.7550	*P* < 0.0001	*P* < 0.0001	*P* < 0.0001

30	48.66 ± 20.37	53.77 ± 16.37	57.92 ± 18.24	8.47 ± 22.43	7.72 ± 24.67	7.82 ± 25.82
	(∗)	(∗)(∗∗)	(∗)(∗∗)			
	*P* = 0.4416	*P* = 0.3155	*P* = 0.6724	*P* < 0.0001	*P* < 0.0001	*P* < 0.0001

60	52.26 ± 20.21	30.01 ± 2.00	57.06 ± 17.07	4.44 ± 15.73	3.52 ± 10.03	3.83 ± 9.23
	(∗)		(∗)			
	*P* = 0.7725	*P* < 0.0001	*P* = 0.8427	*P* < 0.0001	*P* < 0.0001	*P* < 0.0001

120	32.44 ± 26.46	30.76 ± 19.34	35.23 ± 27.32	3.37 ± 10.37	2.81 ± 4.58	3.06 ± 13.34
	*P* = 0.0009	*P* < 0.0001	*P* = 0.0037	*P* < 0.0001	*P* < 0.0001	*P* < 0.0001

180	28.00 ± 19.93	23.85 ± 11.77	31.50 ± 21.51	4.27 ± 5.78	3.77 ± 13.31	3.89 ± 12.2
	(∗)		(∗)			
	*P* = 0.3317	*P* < 0.0001	*P* = 0.5931	*P* < 0.0001	*P* < 0.0001	*P* < 0.0001

**Table 3:** Average Olive moment mean values ± (SD) of the amount of DNA damage of NHDF line before and after leachate treatment. Shapiro-Wilk statistic and its probability for testing distributions normality. Normal distribution before transformation (∗). Normal distribution after log_10_ transformation (∗∗).

Czas (min)	Before treatment			After treatment		
	Olive moment			Olive moment		
	NHDF_0.1%	NHDF_1%	NHDF_10%	NHDF_0.1%	NHDF_1%	NHDF_10%
control_0		1.93 ± 2.63			1.29 ± 1.69	
		(∗∗)				
		*P* = 0.079			*P* < 0.0001	

control_180		2.39 ± 2.31			0.76 ± 1.46	
					(∗∗)	
		*P* = 0.0062			*P* < 0.0001	

0	50.61 ± 29.43	62.16 ± 30.85	67.77 ± 59.17	9.29 ± 19.48	18.5 ± 28.12	37.9 ± 26.16
	(∗)(∗∗)	(∗∗)	(∗∗)			(∗)
	*P* = 0.5852	*P* = 0.0360	*P* = 0.0068	*P* < 0.0001	*P* < 0.0001	*P* = 0.1595

15	47.83 ± 37.91	53.37 ± 32.65	52.25 ± 28.81	4.13 ± 13.83	0.72 ± 1.28	31.79 ± 24.7
	(∗)(∗∗)	(∗)	(∗)(∗∗)			(∗)
	*P* = 0.9238	*P* = 0.6857	*P* = 0.4262	*P* < 0.0001	*P* < 0.0001	*P* = 0.7547

30	43.48 ± 22.37	40.47 ± 27.76	46.40 ± 31.38	1.6 ± 4.99	0.89 ± 1.29	1.9 ± 7.58
	(∗)(∗∗)	(∗∗)	(∗∗)		(∗∗)	
	*P* = 0.6772	*P* = 0.0128	*P* = 0.0386	*P* < 0.0001	*P* < 0.0001	*P* < 0.0001

60	16.29 ± 9.11	46.34 ± 18.11	52.96 ± 47.95	0.76 ± 1.38	0.3 ± 0.8	1.57 ± 5.95
	(∗)(∗∗)	(∗)(∗∗)				
	*P* = 0.8242	0.3361	*P* < 0.0001	*P* < 0.0001	*P* < 0.0001	*P* < 0.0001

120	22.37 ± 9.37	34.55 ± 45.40	45.20 ± 21.99	0.86 ± 1.83	0.38 ± 1.06	1.36 ± 3.74
			(∗)(∗∗)			
	*P* < 0.0001	*P* < 0.0001	*P* = 0.3385	*P* < 0.0001	*P* < 0.0001	*P* < 0.0001

180	14.00 ± 2.47	27.92 ± 20.05	44.98 ± 23.42	0.2 ± 0.46	0.3 ± 1.0	0.28 ± 0.57
	(∗∗)	(∗)(∗∗)		(∗∗)	(∗∗)	
	*P* < 0.0001	*P* < 0.0001	*P* = 0.0164	*P* < 0.0001	*P* < 0.0001	*P* < 0.0001

**Table 4:** Statistical significance calculated with Friedman test (nonparametric analysis of variance).

Log C	Me45				NHDF			
	Before treatment		After treatment		Before treatment		After treatment	
	*P* for *F*-test	*τ*	*P* for *F*-test	*τ*	*P* for *F*-test	*τ*	*P *for *F*-test	*τ*
−1	*P* < 0.0001	0.60	*P* > 0.05	0.03	*P* < 0.0001	0.63	*P* < 0.05	0.07
0	*P* < 0.0001	0.87	*P* < 0.05	0.03	*P* < 0.0001	0.42	*P* < 0.05	0.55
1	*P* < 0.0001	0.32	*P* < 0.05	0.06	*P* < 0.05	0.15	*P* < 0.05	0.67
